# Therapeutics

**Published:** 1877-01

**Authors:** 


### V. Therapeutics.

1. *Liquor Potassæ in Diphtheria.* Shall. (*Boston Jour. Chem.*, June, 1876.)

This agent is considered by the writer the quickest solvent of the diphtheritic membrane. Twenty drops doses every three hours, in the first case in which liquor potassæ was used, after 24 hours' use of iron, potassic chlorate, ammonia, etc., was followed by an entire disappearance of the membrane and by defervescence. Its use is followed by uniformity of result, viz.: disappearance of membrane. While it is not considered a *specific* by the writer, it is declared extraordinarily useful. Dose, 20 drops every three hours, diluted largely, for adults; for minors, dose must be graduated.

2. *Ergot, Hypodermically, in Spleen Enlargement.* Miller. (*Cin. Med. News*, 1876.)

The spleen, in the case reported, enlarged from malarial poisoning, occupied nearly all the abdominal cavity, extending into the right hypochondrium, and from the umbilicus into the epigastrium. Other remedies having uterly failed, ergot, hypodermically daily, reduced the organ to proper proportions in less than 10 days. Nearly a month later the reduction was found to be permanent.

3. *Wash to Prevent Bedsores.* (*New Remedies.*)

For this purpose Sir James Paget recommends a wash composed of one part sweet spirits of nitre, and three parts water. J. S. K.

4. *Calabar Bean as a Galactagogue.* (*New Remedies.*)

In the *Bristol Medical Journal* of Oct. 28th, Dr. W. Monro remarks that he had already brought before the profession various uses to which Calabar bean might be put, from its power of dilating the peripheral blood vessels. Wishing, recently, to restore the secretion of milk after it had disappeared from the breast about three days, he had prepared an ointment of the strength of 20 grains of the bean to the ounce, and ordered it to be applied and washed off carefully before the baby was allowed to suck. After two application, the baby not having been put to the breast in the meanwhile, the milk returned in full flow. J. S. K.

5. *Lime Water in Infantile Eczema and Impetigo.* (*Bulletin de Therap Southern Med. Record.*)

It is especially useful in chronic cases of eczema of head and impetigo of face that have resisted other treatment. It is to be taken in quantities varying up to eight ounces, daily, according to age. If the secretions are very irritating dust the parts with carbonate of magnesia. J. S. K.

6. *Sulphide of Carbon.* DOERING. (*Pacific Med. and Surg. Journal*, Oct., 1876.)

The following brief summary embodies the results the writer has obtained after an extensive trial with this drug:

1. Sulphide of carbon is particularly useful in all ulcers showing a tendency to spread, especially if of a syphilitic nature. It ought to be applied freely at least twice a day.

2. If no beneficial effect is observed after trial with this drug for a week, in any class of ulcer, it will be useless to continue its further application.

3. It is by far the best local application thus far presented to the profession in the treatment of that large class of ulcers termed indolent or chronic.

Sulphide of carbon is a transparent, colorless, exceedingly volatile fluid, of pungent, aromatic taste and very fetid smell. The mode of application is to lightly brush the surface of the ulcer by means of a camel hair pencil or piece of lint, and then cover the surface with some mild, unirritating powder, as subnitrate of bismuth or starch. The application generally produces severe pain, which, however, lasts but a few seconds. J. S. K.

7. *Bromohydrate of Conia.* MOURRUT. (*Répertoire de Pharmacie.*)

M. M. states that this salt has been used with success in the treatment of whooping cough, in doses of one-twelfth grain, if necessary every hour, for a child three years of age; or one-thirtieth grain for a child of one year; or one-sixth grain for adults. Good results have also followed its hypodermic use in sciatica, in quantities of one-twelfth grain. The salt, when pure, occurs in colorless prismatic needles, soluble in water and alcohol, less so in ether or chloroform, is odorless, nearly tasteless and deliquescent. Exposed to the light it turns red but does not decompose, should therefore be kept dark. J. S. K.

8. *Conium.* HAMILTON. (*Med. and Surg. Reporter*, Nov. 4, 1876.)

Dr. H. says that in the treatment of diseases where tremor is a symptom much benefit has followed the use of conium at the Female Epileptic and Paralytic Hospital, Blackwell's Island. In two cases of chorea, of long standing, it produced a prompt amelioration of the patient's condition, and in the tremor of sclerosis it suppressed the movements for several weeks. It was given in the form of fluid extract in doses of 10 m., three times a day. J. S. K.

9. *Ergot in Diarrhœa.* COMEGYS. (*N. Y. Med. Record*, Aug. 26, 1876.)

Dr. C. was induced thus to use this drug from a belief in its power in causing contraction in unstriped muscular fibre. If such were its action it would relieve the atony of the vessels of the intestinal mucous membrane, diminish hyperæmia by contracting the capillaries, and prevent the transudation of the watery part of the blood. To one patient, suffering from chronic diarrhœa of two years standing, 40 drops of fluid ext. ergot were given three times a day, and in four days the number of stools was reduced from eight to two per day. In 10 days the patient was quite recovered. A similar successful use of ergot in many other cases causes him to recommend the treatment to the profession. J. S. K.

10. *Monobromide of Camphor.* GOSS. (*Med. and Surg. Reporter*, Oct. 28, 1876.)

The monobromide of camphor consists of one equivalent of camphor and one of bromine united ($C_{10}$, $N_{16}$, O, Br.). It is a white crystalline salt, having the odor of camphor and slightly that of bromine. The atmosphere decomposes it at a temperature of 100° F. W. A. Hammond has used it successfully in infantile convulsions from teething, dose one gr. each hour; hysteria, four gr. each hour; headache in females from nervous excitement or over study, one dose of four grains being sufficient for cure.

Dr. G. says in chordee, in doses of one or two grains each hour, it is a very positive remedy, one or two doses generally giving relief. In nymphomania there is no remedy equal to this compound salt of camphor and bromine. It is also a positive remedy in spermatorrhœa, nocturnal emissions with amorous dreams, in doses of three or four grains, at bedtime.

In cases of cerebral anæmia, from excessive venery, it calms nervous excitement; in debility, with cold extremities from feeble heart, it equalizes circulation—impressing the cerebro-spinal system. Dose, three or four grains three times a day.

In nocturnal incontinences of urine it is efficacious in doses of from one to six grains at bedtime.

(Best given disolved in alcohol and glycerine, or suspended in mucilage and syrup as it irritates the stomach—J. S. K.)

11. *Viburnum Primifolium.* JENKS. (*N. Y. Med. Record.*)

The virtue attributed to black haw is that it prevents abortions by some sedative action upon the uterus, apparently the opposite of that of ergot. It has been used extensively for this purpose by Drs. Faris, of Miss., Jenks, of Detroit, and Bates, of New York. It is also useful in menorrhagia coming on during the menopause, and in cases of dysmenorrhœa when there is no mechanical cause of obstruction.

The method of administration is to give the fluid extract in doses of from one-half to one drachm a few days before and a few days after the menstrual period, or from two to four grains of resinoid in the same manner. J. S. K.

12. *Bromide of Potassium as a Caustic.* PEYRAND. (*Canadian Journal of Med. Science.* Nov., 1876.)

M. Peyrand's first clinical experiment on the subject took place in April, 1874, when by means of daily applications of powered bromide he effected the removal, within 28 days, of an epitheliomatous growth on the face. He has since had equally good results from this treatment of atonic ulcers of the legs; rapid cicatrization following the separation of sloughs following the application. In such cases he uses either the powder or an ointment of one part in five, or a mixture (one in ten) of glycerine. In many skin affections, as chronic eczema, pityriasis, and acne, in phagedæna, ulcerative stomatitis, and many other local inflammatory disorders, he has found it of use. As a local hæmostatic a solution of one part in fifty has served for epistaxis; and as a general hæmostatic its success in many cases of hæmoptysis and metrorrhagia was very marked where ergot, perchloride of iron and rhatany had failed. J. S. K.

